# Clinical Screening for Congenital Heart Disease in Newborns at a Tertiary Care Hospital of a Developing Country

**DOI:** 10.7759/cureus.4808

**Published:** 2019-06-03

**Authors:** Muhammad Mohsin, Khadija N Humayun, Mehnaz Atiq

**Affiliations:** 1 Pediatrics, Aga Khan University Hospital, Karachi, PAK; 2 Paediatrics & Child Health, Aga Khan University Hospital, Karachi, PAK

**Keywords:** congenital heart disease, pulse oximetry screening, critical congenital heart disease

## Abstract

Objective: To screen all newborns admitted to a tertiary care hospital to rule out congenital heart disease before discharge and to find out the utility of pulse oximetry to detect congenital heart disease.

Methodology: This prospective study was done at Aga Khan University Hospital from January 2014 to December 2014 in 1,650 newborns over a period of 12 months. Pulse oximetry and clinical examination were done. Persistent oxygen saturation less than 95% was considered as positive pulse oximetry. Newborns who had positive pulse oximetry or abnormal clinical examinations findings were subjected to echocardiography.

Results: Pulse oximetry was performed on 1,650 newborns, out of which 25 (1.5%) had congenital heart disease. Positive pulse oximetry cases were 16 (0.97%), out of which 10 had only positive pulse oximetry (negative clinical examination). Positive clinical examination cases were 45 (2.7%), out of which 39 cases had only positive clinical examinations (negative pulse oximetry). Six newborns had both positive pulse oximetry and positive clinical examination. Out of the 25 diagnosed cases of congenital heart disease, ventricular septal defect (VSD) was the most common congenital heart disease, followed by patent ductus arteriosus (PDA). The sensitivity, specificity, positive predictive value, and negative predictive value of pulse oximetry were 32%, 99.5%, 50%, and 98.9% respectively.

Conclusion: In the community setting of a developing country, a combination of pulse oximetry screening and clinical examination are better at detecting congenital heart defects than either test alone.

## Introduction

Congenital heart disease (CHD) is the commonest group of congenital malformations, affecting 7 to 8 out of every 1,000 newborns [1]. CHD accounts for 6% - 10% of all infant deaths and accounts for 20% - 40% of all infant deaths that occur due to malformation [2]. One of the major contributors to increased infant mortality and morbidity is the clinical deterioration and collapse prior to diagnosis and treatment [1]. Congenital heart defects involve a problem during the development of the heart which can manifest at any age. This problem can be mild with no significant hemodynamic compromise to critical, requiring early intervention and surgeries. About 25% of CHDs are life-threatening and may manifest before the first routine clinical examination [2-3]. Failure to identify these critical lesions immediately after birth leads to a delay in referral and increased mortality and morbidity [4]. Many studies have shown that the measurement of oxygen saturation identifies neonates with mild cyanosis who do not have an audible murmur or other signs of cardiac abnormality and are not detected by routine clinical examination [5]. Combining pulse oximetry with clinical examination can enhance the clinician’s ability to detect life-threatening CHD in a timely manner [6-7]. The American Heart Association (AHA) has recommended future studies across a broad range of delivery systems to determine whether this practice should become the standard of care in the routine assessment of neonates [8]. Recent studies reported high sensitivity and specificity of pulse oximetry screening in newborns to detect congenital heart disease [9-10]. We carried out the study with the objective of 1) screening all newborns admitted in the nursery and neonatal intensive care unit (NICU) to rule out congenital heart disease before discharge from hospital and 2) to find out the utility of pulse oximetry to detect congenital heart disease.

## Materials and methods

This hospital-based prospective study was conducted at the Aga Khan University Hospital, Karachi from January 2014 to December 2014. All newborns admitted to the Well Baby Unit and NICU during the study period were enrolled after taking informed consent from the parent. All those newborns who had gestational age < 34 weeks, birth weight < 1.5 kg, discharge before 48 hours of life, and those babies who were very sick, on a mechanical ventilator, and in whom stable pulse oximeter signals could not be obtained were excluded from the study. The study protocol was approved by the Ethics Review Committee of Aga Khan University.

Oxygen saturation (SpO_2_) measurements of all neonates were recorded by registered nurses using the Ohmeda 3700 pulse oximeter. For each newborn, oxygen saturation at birth, 24 hours, and 48 hours of life were obtained. The pulse oximeter probe was applied on either foot. The recordings were noted after obtaining stable pulse oximeter signals. A SpO_2_ of ≥ 95% was defined as negative pulse oximetry screening (POS). POS was positive when a SpO_2_ of ≤ 94% was documented and it was rechecked after one hour. If still positive, it was followed by echocardiography. If the POS was negative, no further workup was done. Clinical examination screening was performed for all newborns with special reference to signs and symptoms related to the cardiovascular system to detect congenital heart disease. Presence of central cyanosis, heart murmurs, abnormal peripheral pulses, abnormal precordium, and tachypnea were taken as positive clinical examination findings suggestive of congenital heart disease [9]. The newborns with a positive clinical exam or positive pulse oximetry underwent echocardiography. In case of detection of any abnormality, the type of abnormality detected was also recorded and the parents were counseled regarding appropriate management. For the normal babies, a follow-up evaluation (clinical evaluation or telephonic interview of parents) was performed at six weeks. If this was abnormal, echocardiography was repeated. Collected data were analyzed using the Statistical Package for Social Sciences (SPSS), version 20.0 (IBM SPSS Statistics, Armonk, NY). Sensitivity, specificity, positive predictive value, a negative predictive value of pulse oximetry, and clinical examination were calculated and interpreted accordingly.

## Results

**Figure 1:**
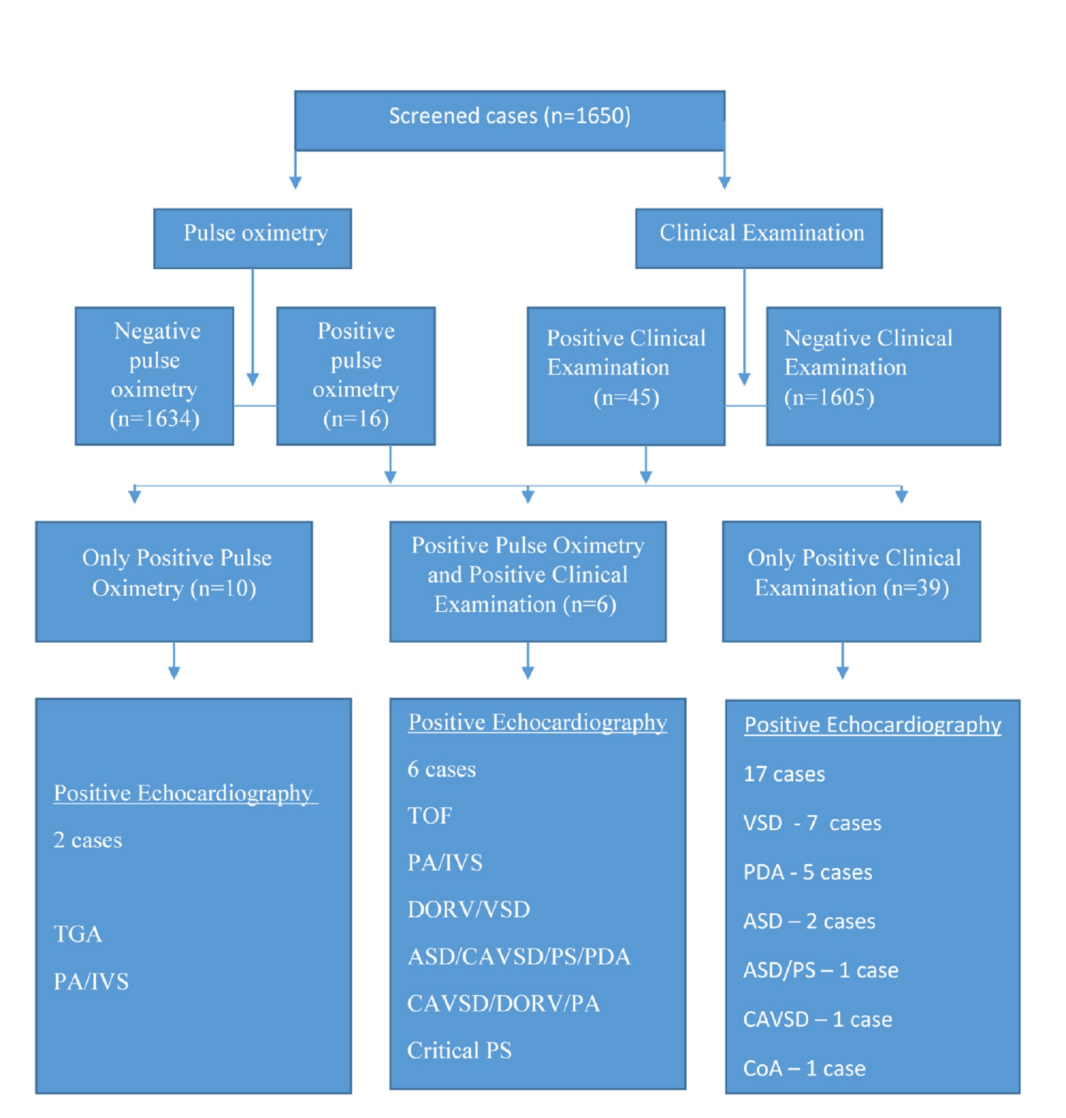
Details of pulse oximetry screening and clinical examination of the newborns under study ASD: atrial septal defect; CAVSD: complete atrioventricular septal defect; CoA: coarctation of aorta; DORV: double outlet right ventricle; IVS: intact ventricular septum; PA: pulmonary atresia; PDA: patent ductus arteriosus; PS: pulmonary stenosis; TGA: transposition of great arteries; TOF: tetralogy of Fallot; VSD: ventricular septal defect

A total of 1,650 neonates were enrolled in the study. Pulse oximetry screening and clinical examination were performed on all newborns during the study interval. Nine hundred and twenty-four newborns (56%) were females and 726 (44%) were males. The mean age was 38.9 weeks. Out of the 1,650 screened cases for pulse oximetry, 16 (0.97%) had positive pulse oximetry. On the other hand, on clinical examination, 45 (2.7%) newborns had positive findings suggestive of congenital heart disease. Six newborns were found to be positive for both pulse oximetry and clinical examinations. In this study, 10 newborns only had positive pulse oximetry and 39 only had a positive clinical examination. On echocardiography of all 55 positive cases, we found 25 newborns had congenital heart disease (Figure 1).

All six cases who had positive pulse oximetry and positive clinical examination had congenital heart disease. Two newborns had congenital heart disease out of 10 positive pulse oximetry cases without positive clinical examination. Out of eight false positive cases for pulse oximetry screening, six patients were diagnosed with persistent pulmonary hypertension and two with congenital pneumonia. Out of 39 cases with positive clinical examination without positive pulse oximetry, 17 had congenital heart disease. Two patients presented with asymptomatic murmur on follow-up; an echocardiogram showed physiological branch pulmonary stenosis and small muscular ventricular septal defect (VSD). Both cases were considered as normal because they were expected to improve with age and required no follow-up. The total congenital heart disease cases detected in our study was 25 (1.5%) out of 1,650 newborns. The sensitivity, specificity, positive predictive value, and negative predictive value of pulse oximetry were 32%, 99.5%, 50%, and 98.9%, respectively (Table 1).

**Table 1:** Sensitivity, specificity, positive predictive value, and negative predictive value of pulse oximetry screening and clinical examination

	Pulse oximetry (POS)	Clinical Examination (CE)
Sensitivity	32%	92%
Specificity	99.5%	98.6%
Positive predictive value	50%	51.1%
Negative predictive value	98.9%	99.8%

We found VSD was the most common congenital heart disease, followed by patent ductus arteriosus (PDA), atrial septal defect (ASD), and pulmonary atresia (PA) with an intact ventricular septum (IVS) in this study.

## Discussion

Diagnosis and treatment of congenital heart disease have changed dramatically over the last few decades. The relative importance of preoperative morbidity and mortality has been increasing as a result of the great improvements in perioperative and long-term survival. In our study, 25 (1.5%) newborns were detected to have congenital heart disease out of total 1,650 screened cases. Among the congenital heart disease diagnosed, VSD was the most common congenital heart disease, followed by PDA, ASD, and PA with an IVS. Kalita et al. found 34 (1.98%) cases with congenital heart disease in their study of 1,720 newborns [11]. However, another study conducted by Mathur et al. detected 72 (7.57%) cases with congenital heart disease out of 950 screened cases [12], which was much higher than in our study. Our finding is comparable to the study by Kalita et al. [11].

This study highlights some restrictions of only POS in developing countries. No prior study applying such methodology has been carried out in Pakistan. The present study reviews the sensitivity, specificity, positive predictive value, and negative predictive value of pulse oximetry screening and clinical examination. In our study, we found the sensitivity, specificity, positive predictive value, and negative predictive value of POS to detect CHD were 32%, 99.5%, 50%, and 98.9%, respectively. Vaidyanathan et al. [13] from Kerala observed 11.4% sensitivity and 90.9% specificity for POS, which was low. Kalita et al. reported the sensitivity of pulse oximetry was 41.1%, which was similar to our findings [11]. However, many other authors reported a high sensitivity of POS; one, in particular, carried out in Delhi by Mathur et al., observed the sensitivity of pulse oximetry up to 95.2% in their study [12] but they included patients with persistent pulmonary hypertension (PPHN). If we included patients with PPHN as congenital heart disease sensitivity in our study, it also increases to 82.3%. Although PPHN has been considered as a secondary target of pulse oximetry and echocardiography, it is recommended but not considered as congenital heart disease. In this study, we found a high sensitivity (92%) and specificity (98.6%) of clinical examination which are comparable with the results observed by Kalita et al. having a sensitivity (82.3%) and specificity (99.5%) [11]. Some studies reported unsatisfactory results of clinical examination to detect congenital heart disease which might be due to the different levels of expertise of the clinicians involved to diagnose congenital heart disease. In our study, six newborns were found to be positive for both pulse oximetry and clinical examination. All six cases had congenital heart disease (100%). Therefore, a combination of clinical exam and pulse oximetry screening will help in the detection of congenital heart disease.

The findings of our study suggested that the benefits of POS shown in high-income countries could be translated to low-income developing countries (like Pakistan) with great success, but clinical examination should be included as part of the screening to detect congenital heart disease.

## Conclusions

The combination of POS and clinical examination is better at detecting congenital heart defects than either pulse oximetry or clinical examination alone. By detecting congenital heart defects early, infants can often be treated in a timely fashion and lead longer healthier lives.
